# *In Vitro* Calcification of Bioprosthetic Heart Valves: Test Fluid Validation on Prosthetic Material Samples

**DOI:** 10.1007/s10439-020-02618-6

**Published:** 2020-09-28

**Authors:** N. Kiesendahl, C. Schmitz, M. Menne, T. Schmitz-Rode, U. Steinseifer

**Affiliations:** 1 Department of Cardiovascular Engineering, Institute of Applied Medical Engineering, Helmholtz Institute Aachen, RWTH Aachen University, Pauwelsstraße 20, 52074 Aachen, Germany; 2 Institute of Applied Medical Engineering, Helmholtz Institute Aachen, RWTH Aachen University, Aachen, Germany; 3 ac.biomed GmbH, Aachen, Germany

**Keywords:** Fluid validation, Spontaneous precipitation, Intrinsic calcification, Structural identification

## Abstract

Calcification is a major failure mode of bioprosthetic heart valves. So far, cost and time saving *in vitro* analyses of calcification potentials are unreliable, mostly due to superficial or spontaneous precipitation of the applied fluids. In this study, we developed a near-physiological non-spontaneously precipitating fluid for an accelerated *in vitro* calcification assessment, and validated it by analyzing the calcification potential of two prosthetic materials within two reference-tests. The first test focused on the comparison of four calcification fluids under dynamic contact with n=12 commercial bovine pericardium patches. The second one focused on the validation of the most appropriate fluid by analyzing the calcification potential of pericardium vs. polyurethane. The patches were mounted in separate test compartments and treated simultaneously with the respective fluids at an accelerated test frequency. Calcification propensity and progression were detected macroscopically and microscopically. Structural analyses of all deposits indicated hydroxyapatite by X-ray powder diffraction, which is also most commonly observed *in vivo*. Histological examination by von Kossa staining showed matrix internal and superficial calcifications, depending on the fluid composition. The present study reveals promising results towards the development of a meaningful, cost and time saving *in vitro* analysis of the calcification potential of bioprosthetic heart valves.

## Introduction

Calcification is a major reason for the failure of bioprosthetic heart valves.^26^ Usually, the calcification propensity of certain materials as well as potential anti-calcification treatments are assessed in animal studies. Large animal studies are costly and time-consuming, and studies on small animals are limited to subcutaneous implantation, thus disregarding the direct exposition to the circulatory blood system and the influence of mechanical stress on the calcification behavior.^26^,^33^ Therefore, several attempts towards an accelerated *in vitro* model were undertaken in order to provide a cost- and time-saving method for the analysis of calcification processes.^1^,^3^,^10^,^13^,^14^,^16^ Due to the problem of superficial or spontaneous precipitation, which occurred in the fluids former applied, we performed a preliminary fluid study on the development of a near-physiological calcification fluid.^13^ The desired fluid should not precipitate spontaneously and should neither promote nor inhibit calcification. Eleven different fluid compositions were tested without contact to potentially calcifying materials, firstly to investigate their potential for homogeneous nucleation and thus spontaneous precipitation.^13^ For the development of a suitable *in vitro* test method for the calcification potential of bioprosthetic heart valves, both forms of nucleation, homogeneously and heterogeneously, are important factors. The heterogeneous nucleation represents the material-induced calcification, a relevant parameter for the comparison of different valve materials, which can occur intrinsically within the materials’ matrix and on its surface. Homogeneous nucleation, on the other hand, occurs independently from the presence of any test substrate, and will thus lead to diffuse and primarily superficial calcification, which does not permit an objective comparison of different materials. To avoid this form of calcification in the development of a suitable in-vitro test method, we deliberately focused our previous fluid study on the development of non-spontaneous precipitating fluids. In the present study, we now investigated the heterogeneous calcification tendency of the non-spontaneous precipitating fluids in contact with potentially calcifying materials under dynamic conditions. Additionally, we now investigated two spontaneously precipitating fluids of our preliminary fluid study^13^ under dynamic material-contact in order to compare the crystalline structure of substrate-surface-induced calcifications vs. spontaneously precipitated deposits with the crystal structures of calcifications found *in vivo*. The most common calcium phosphate phase, which occurs *in vivo*, is hydroxyapatite (HAP). According to Elliot,^8^ HAP represents the thermodynamically most stable phase of the three phases dicalciumphosphate-dihydrate (DCPD), octacalcium phosphate (OCP) and HAP at pH 7.4 and 37 °C. It can be assumed that the precipitation of HAP proceeds from DCPD *via* OCP as a metastable precursor during the recrystallization process. 3,12,13,18

In the present study, we conducted two reference tests. The first one focused on the comparison of the precipitation behavior of four different fluids (two non-spontaneously precipitating fluids, one fluid with low spontaneous precipitation tendency producing OCP as main crystal phase and one spontaneously precipitating fluid producing HAP)^13^ under dynamic contact with bovine pericardium patches that have been pretreated in the same way by the manufacturer. The calcification analysis included the initiation, progression, and generation of crystal phases of calciumphosphate and the localization of the calcifications (superficial or intrinsic). The second reference test was based on the results of the first test and focused on the assessment of the most appropriate fluid in dynamic contact with two different patch materials (bovine pericardium and polyurethane) concerning its potential to reveal differentiated material propensities. The mechanism for polyurethane calcification is not yet completely understood. Generally, it is postulated as surface phenomenon, depending on roughness, porosity and defects like cracks, fractures and micro-gaps. All these factors are considered responsible for an (increasing) calcification propensity of PU. This can take place by deposition and attachment of cell debris and thrombi, as well as trapping of calcium-binding serum proteins and phospholipids to serve as nucleators, or the mere deposition of calcium phosphate compounds from a precipitating solution. The hypothesis of calcium diffusion *via* pores and penetration of the calcification to the subsurface is considered controversial. In addition, the role of mechanical stress as a contributor to calcification is seen controversially. The bond strength between the calcified layer and the polyurethane surface is described as very weak, approximately in the range of Van-der-Waals forces.^31^,^32^ In contrast, tissue calcification is postulated as a subsurface phenomenon.^31^,^32^ For bioprostheses a non-energy-requiring Ca^2+^ trap mechanism by phospholipids or due to residual cell fragments that have been devitalized by glutaraldehyde treatment is most probably responsible for the calcification.^3^ Furthermore an active involvement of the pericardial collagen textures and residues of charged sidechains in the initiation, orientation and calciumphosphate phase formation is considered.^17^,^29^

All tests were accompanied by the structural analysis of the resulting deposits and histological localization of the calcification within the tested material or onto its surface.

## Materials and Methods

### Fluid-Reference-Test

The calcification propensity of four differently composed fluids was tested under accelerated frequency and physiological pressure loads on bovine pericardial patches.

#### Chemicals

All chemicals used were of analytical grade. The water used for the calcification solutions was sterile double-distilled water.

#### Patches

Twelve commercial glutaraldehyde crosslinked bovine pericardium patches (Edwards bovine pericard 4700) were tested using four different fluids (three patches per fluid; *n* = 3). All patches were of the same order number, of the same pretreatment by the manufacturer and of similar geometry (circular, 38 mm diameter and 0.5 mm thickness).

#### Test System and Settings

The in vitro calcification assessment was conducted in a heart valve durability tester CVE-FT2, a proprietary tester of the Department of Cardiovascular Engineering.^6^,^7^,^13^ The patches were clamped between rigid PVC fixation rings with an outer diameter of 38 mm and an inner diameter of 16 mm and mounted into separate test compartments filled with four different ionic calcification fluids at physiologic temperature of 37 °C and a pH-value of 7.4. All patches had the same resulting fluid contact area of 201 mm^2^. Before assembly, the patches were rinsed with distilled water. In order to ensure comparability with prospective calcification tests on heart valve prostheses, the test conditions were kept consistent with normotensive conditions as specified for durability testing of heart valve prostheses in ISO 5840. Accordingly, the patches were loaded sinusoidally with a peak differential pressure of at least 100 mmHg, which was maintained for 95 % or more of all test cycles and for at least 5 % of the duration of each cycle.

Test frequency was 300 bpm. The test duration was 4 to 9 weeks, depending on the observed results, with a weekly change of the calcification fluid and weekly measurements of the ionic concentrations. To observe the precipitation tendency of the fluids themselves (homogeneous nucleation) without contact to a potentially calcifying substrate, a part of each fluid was kept as a reference in a separate polyethylene (PE) bottle within the durability tester at 37 °C. Thus, the reference fluids were exposed to slight vibrations comparable to the ones within the compartments.

#### Calcification Solutions

The compositions of the investigated calcification solutions based on the preliminary fluid study^13^ with the following parameters (Table 1):

**Table 1:** Investigated fluid compositions.

Fluid	[CaCl_2_] mM	[Ca-Gluc] mM	[Ca_T_] × 10^−3^ M	[KH_2_PO_4_] mM	[NaH_2_PO_4_] mM	[Na_2_HPO_4_] mM	[P_T_] × 10^−3^ M	[NaCl] mM	[KCl] mM	Phase	*S*_CaP_
C	1.5	0.1	1.6	1.25	0.43	0.14	1.8	114	3.8	DCPD OCP HAP	0.05 0.78 × 10^2^ 1.99 × 10^11^
E	2.6	–	2.6	1.0	–	–	1.0	115	4.0	DCPD OCP HAP	~ 0 0.84 × 10^2^ 3.57 × 10^11^
F	1.3	–	1.3	1.0	–	–	1.0	115	4.0	DCPD OCP HAP	− 0.51 5.64 1.40 × 10^10^
G	1.7	–	1.7	1.0	–	–	1.0	115	4.0	DCPD OCP HAP	− 0.39 0.16 × 10^2^ 4.55 × 10^10^
L	2.1	–	2.1	1.0	–	–	1.0	115	4.0	DCPD OCP HAP	− 0.24 0.38 × 10^2^ 1.32 × 10^11^

All selected fluids had an ionic strength of ~ 0.16 M,^11^,^13^,^22^ as assumed for blood plasma, but differed in their Ca_T_ concentrations, and fluid C additionally in the P_T_ concentration. For the same ionic strength, this resulted in different activities of free calcium and HPO_4_^2-^, which allowed the different ion products and saturation levels to be adjusted, especially with regard to the solubility product of DCPD. As the saturation degree concerning the solubility product of DCPD is considered as an initial factor of *in vivo* calcification and spontaneous precipitation,^9^,^18^ the fluids were selected here in such a way that their ion products were more or less far below or just above the solubility product of DCPD. Fluid L was a modification of fluid F, which replaced fluid F after a test duration of 4 weeks, to achieve an accelerated calcification. It was still undersaturated with respect to the Ksp of DCPD, but the [Ca_T_] was increased in order to increase the ionic product towards the Ksp of DCPD and thus decrease the degree of undersaturation towards saturation.

The fluid preparation was performed at room temperature due to a better laboratory handling. Thus the pH adjustment of the barbital buffer by addition of 1N hydrochloric acid solution also took place at room temperature, considering the temperature dependency of the barbital buffer.^13^

Preservation of the calcification fluids during the test period was done by addition of 0.05 % sodium azide to each fluid. Due to the photosensitivity of the barbital buffer, the fluid preparation as well as the calcification testing were performed under light-protected conditions.

#### Analysis

The initial concentrations of total calcium and total phosphate in the fluids were determined by colorimetry (Vitros Chemistry System 350, Ortho-Clinical Diagnostics). The calcification propensity and progression during the test were detected macroscopically and microscopically on the patches´ surfaces. Additionally, the course of the test was controlled by colorimetric measurement of the calcium and phosphate decrease within the fluid of each test compartment. The sodium and potassium concentrations of the fluids were determined potentiometrically, both with the Vitros Chemistry System 350 (Ortho-Clinical Diagnostics).

Structural analysis of the deposits was carried out by X-ray powder diffraction (XRD) according to the availability with a Philips X’Pert Pro (MILIDI) powder diffractometer between 10° and 80° 2*θ* at a sweep rate of 0.1° 2*θ*/min or with a D8 Advance Bruker AXS powder diffractometer between 10° and 80° 2*θ* at a sweep rate of 0.1° 2*θ*/min. When using the D8 device, the sample material was fixated on the sample carrier with silicone paste. The noise component of the signal caused by the silicone paste was determined by taking a reference spectrum of silicone paste, under the same conditions as the actual sample measurements. For XRD measurement, the deposits were mostly separated from the pericardial material, dehydrated with ethanol 98%, suction filtered and dried in a desiccator.

Histological examinations of the calcified pericardium patches were made from 5 µm thin slices of the cross section areas by HE-, EvG-, Alizarin red- and von Kossa staining. The histological examinations were conducted at the immunohistochemistry facility of the IZKF of the medical faculty at RWTH Aachen University.

### Fluid-Material-Differentiation-Test

The calcification propensity of glutaraldehyde crosslinked bovine pericardium patches was tested against polyurethane patches under accelerated frequency and physiological pressure loads using the most appropriate calcification fluid of the Fluid-Reference-Test, fluid L.

#### Chemicals

For the chemicals used here, the same specifications apply as described for the Fluid-Reference-Test.

#### Patches

Three commercial glutaraldehyde crosslinked bovine pericardium patches (Edwards bovine pericard 4700, cf. Fluid-Reference-Test) were tested against three polyurethane (CarbothaneTPU PC-3585A, Lubrizol) patches produced in-house. All patches were of a similar geometry (circular, 38 mm diameter and 0.5 mm thickness).

#### Test System and Settings

The used test system, the mounting of the test patches and the adjusted test conditions were the same as described in the Fluid-Reference-Test. But for the Fluid-Material-Differentiation-Test the same fluid composition was used for all test patches and the test duration of 9 weeks.

#### Calcification Solution

The composition of the calcifying solution used was similar to fluid L of the Fluid-Reference-Test (Table 1).

Fluid preparation, preservation and testing were performed according to the same protocol as described for the Fluid-Reference-Test.

#### Analysis

Fluid concentration measurements and the detection of propensity and progression of the calcifications on the patch surface were performed as described for the Fluid-Reference-Test.

Structural analysis of the deposits was carried out by X-ray powder diffraction (XRD) with a D8 Advance Bruker AXS powder diffractometer between 10° and 80° 2*θ* at a sweep rate of 0.1° 2*θ*/min. The sample material was fixated on the sample carrier with vaseline. The noise component of the signal caused by the vaseline was determined by taking a reference spectrum of vaseline, under the same conditions as the actual sample measurements. For XRD measurement, the deposits were mostly separated from the patch material, dehydrated with ethanol 98 %, suction filtered and dried in a desiccator.

The histological examination of the calcified patches was carried out in the same way as described for the Fluid-Reference-Test.

## Results

### Fluid-Reference-Test

The reference samples of the fluids C and E showed a slight precipitation within the PE bottles during each week. The reference samples of the fluids F and G showed no precipitation.

**FIGURE 1:**
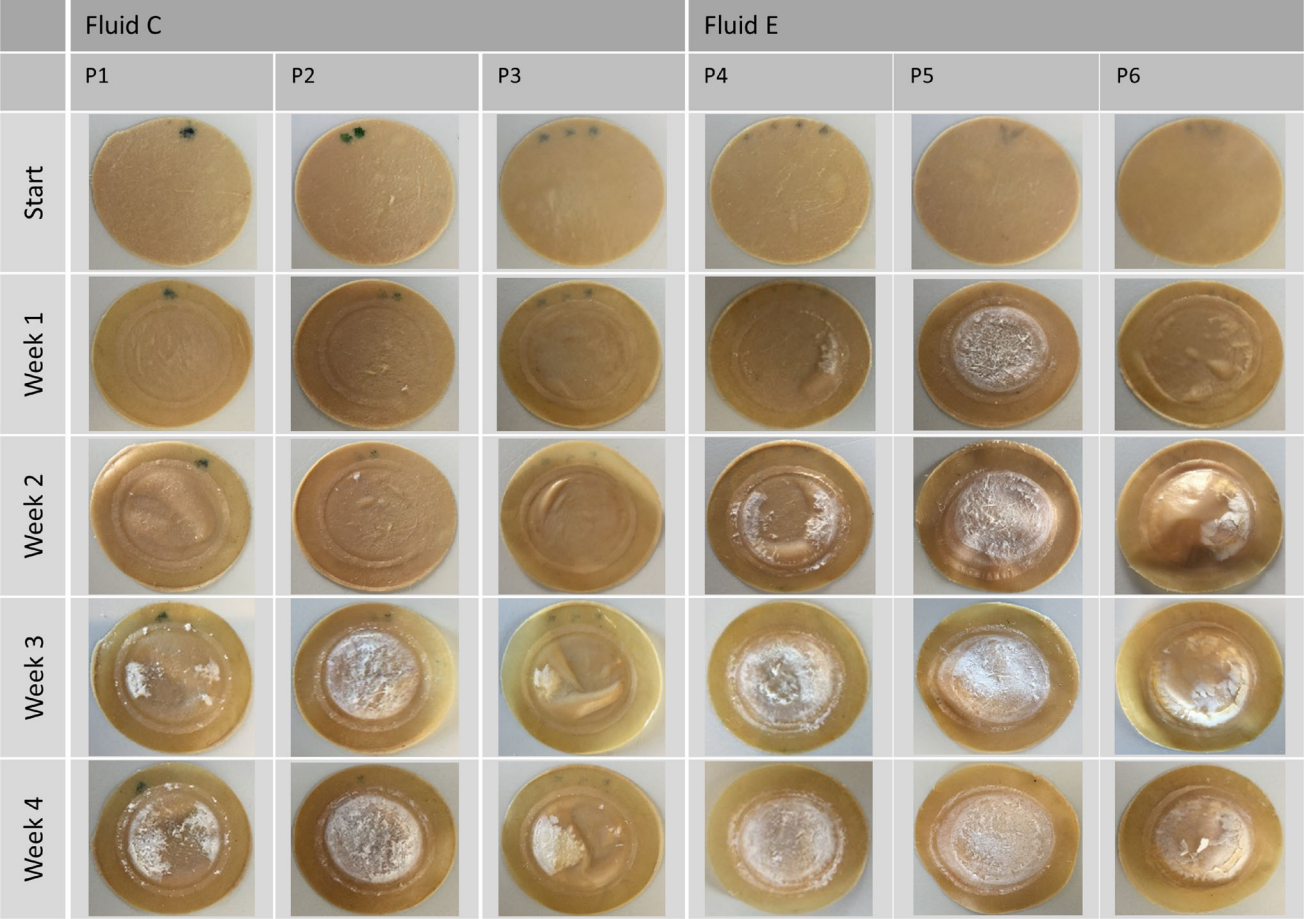
Progression of in vitro calcification of pericardium patches conducted in fluids C (P1–P2) and E (P4–P6) for 4 weeks.

In contact with potentially calcifying pericardium patches the fluids C and E revealed a progressing calcification over 4 weeks (Fig. 1), which started macroscopically in the second or first week, respectively. After 4 weeks, the test with these two fluids was terminated due to the increasing stiffening of the calcified areas of the patches and the flaking of deposits.

**FIGURE 2:**
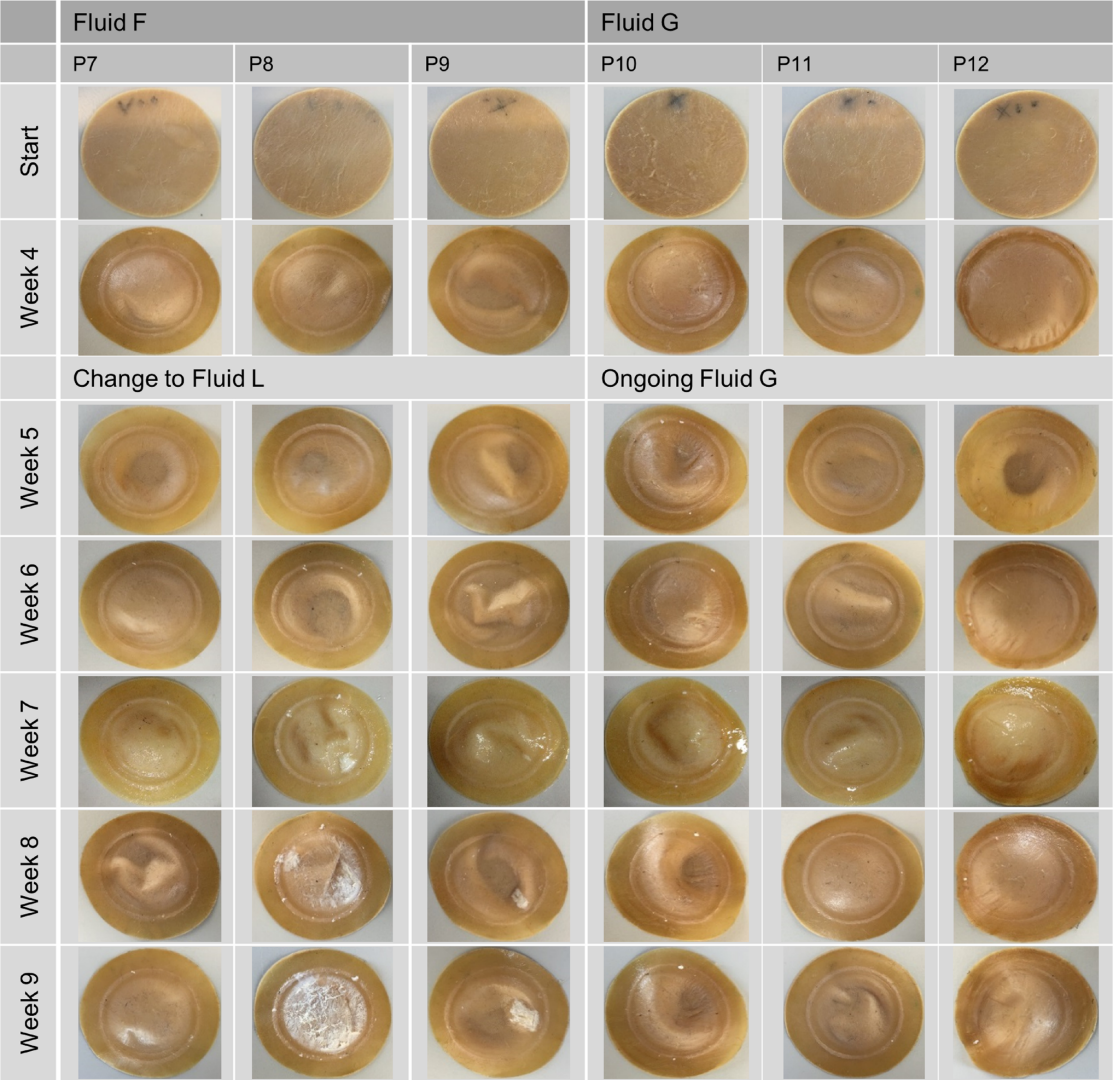
Progression of in vitro calcification of pericardium patches conducted in fluids F (P7–P9), G (P10–P12) and L (P7–P9) for 9 weeks.

The patches tested in the fluids F and G showed no calcification within a testing period of 4 weeks. Thus, the test period with fluid G was prolonged up to 9 weeks. Fluid F, as the most undersaturated fluid, was replaced by its modification, renamed fluid L (Table 1) up from week 5 to week 9, in order to increase the calcification potential. Fluid G was retained in its original concentration and thus represented the lowest concentrated fluid from week 5 onwards. The reference samples of fluid G and L exhibited no precipitation within the PE bottles. The pericardium patches, which were continuative tested within fluid L, started to calcify 2 weeks after the change from fluid F to L (6 weeks of total test duration) and revealed an increasing calcification during the following 3 weeks. Fluid G showed only scattered spots of calcification on the patches even after 9 weeks of testing (Fig. 2).

All calcified pericardium patches showed the beginning of calcification right next to the circular line of fixation, independent of the fluid used. Radial inwards growth of the deposits was observed during the following weeks (Figs. 1 and 2).

#### Structural Analysis of the Deposits

##### XRD

**FIGURE 3:**
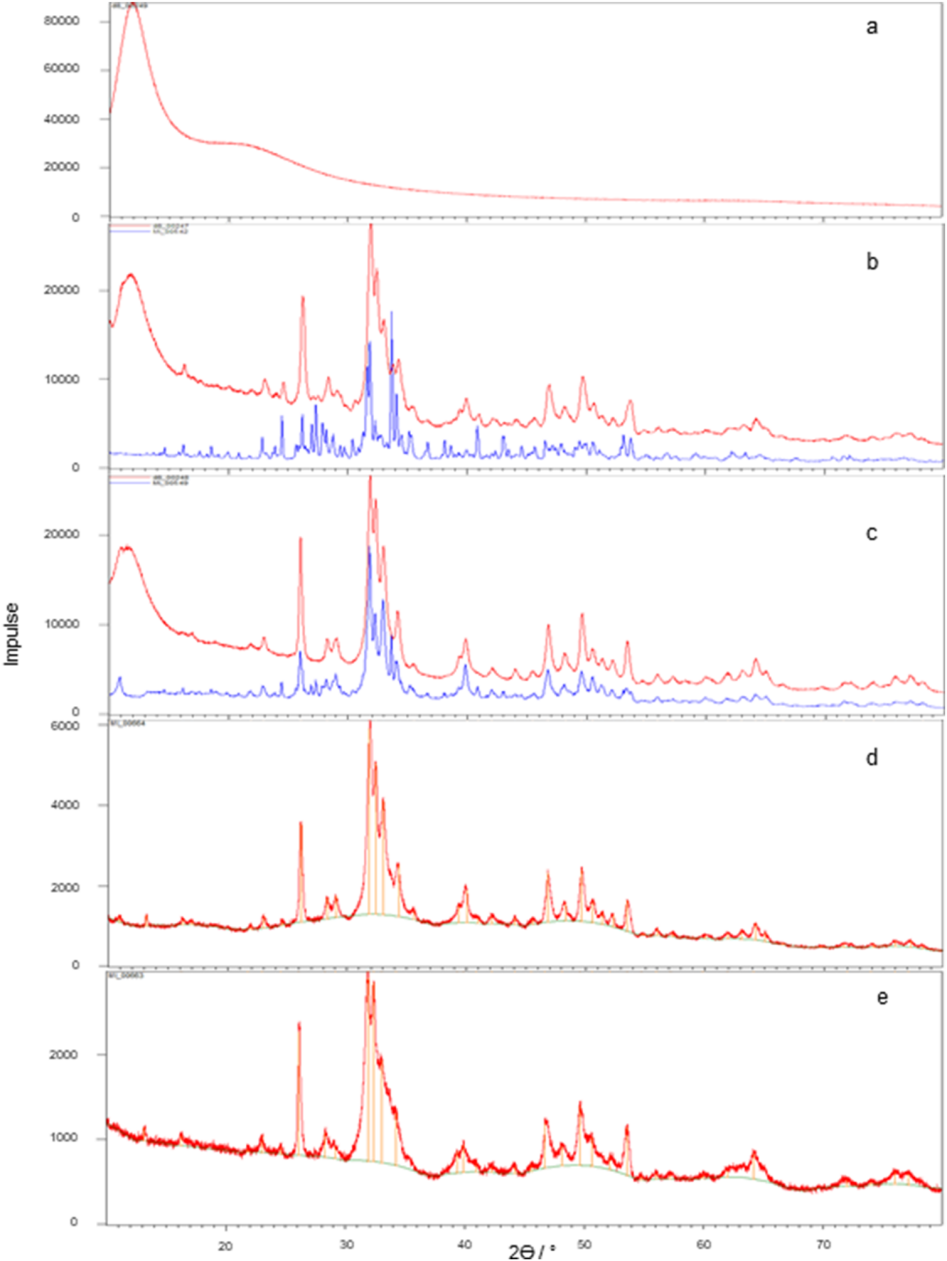
X-ray powder diffractograms of the deposits of the investigated pericardium patches and the precipitates of the corresponding spontaneously precipitating fluids out of the preliminary fluid study.^13^ Silicone paste (a). Deposits of P2 (red line) and the corresponding fluid C (blue line) (b).Deposits of P6 (red line) and the corresponding fluid E (blue line) (c).Deposits of P8 (d). Deposits of P9 (e).

After four (fluids C and E) or nine (Fluid L) weeks of testing, respectively, the resulting deposits from the patches were analyzed by XRD. The patches P10 to P12, tested in fluid G, did not reveal sufficient deposits for analysis. Additionally to the diffractograms of the deposits of the patches P2 and P6 (Figs. 3b + 3c red lines), the diffractograms of the corresponding fluid-precipitates out of the preliminary fluid study^13^ (Figs. 3b + 3c blue lines) are depicted. Furthermore the reference spectrum of silicone paste is shown (Fig. 3a) to determine the noise component of the signals of the diffractograms of the patch-deposits of P2 and P6. For patches P8 and P9 only the diffractograms of the patch deposits are depicted because the corresponding fluid L did not show any spontaneous precipitation. The diffractograms of all patch-deposits show similar peak patterns.

#### Histological Examination

**FIGURE 4:**
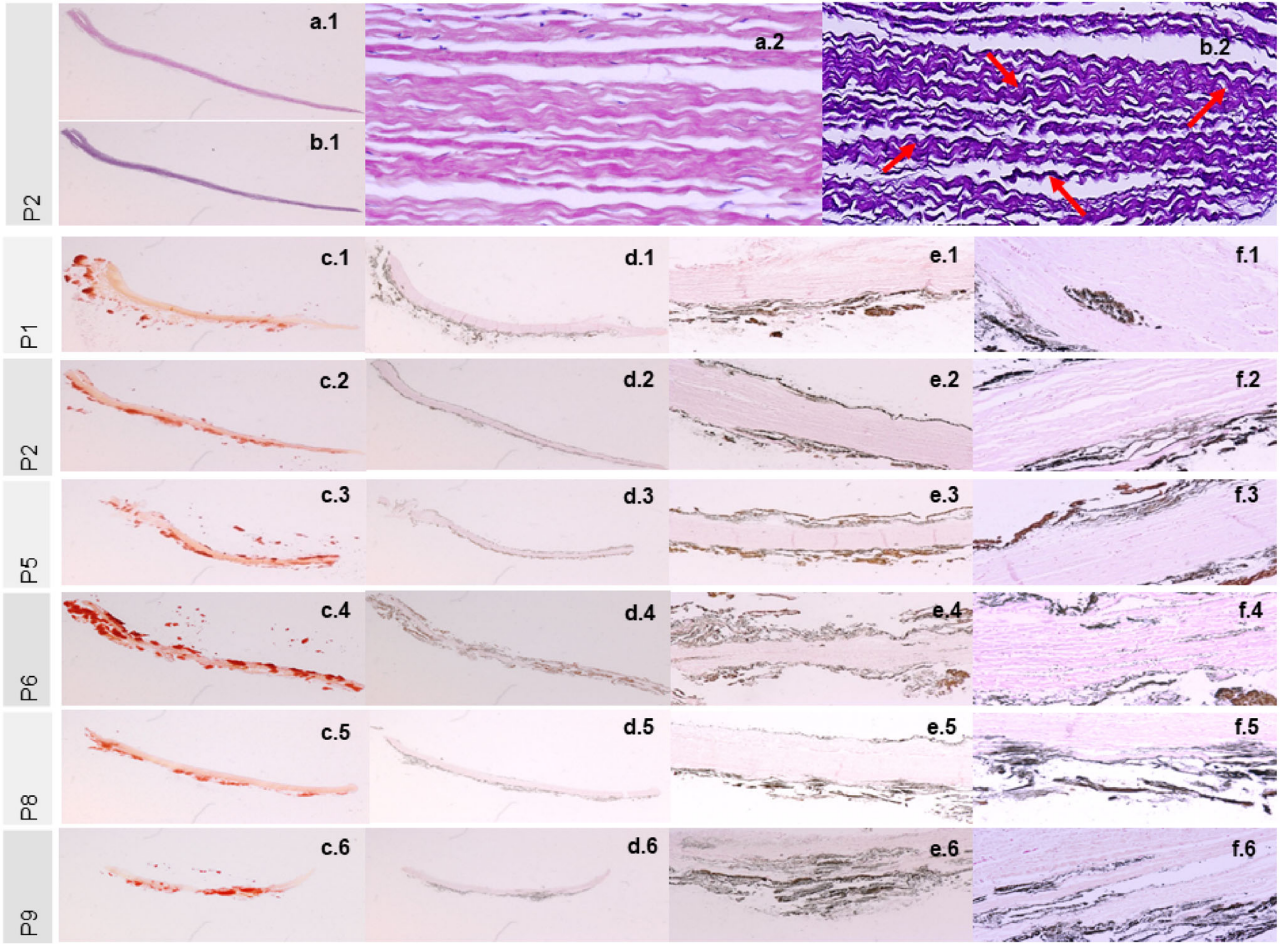
Principal tissue stains HE (a.1 + a.2) and EvG (b.1 + b.2) of cross sections of P2 with increasing magnifications: x  30 (a.1 + b.1) and x 500 (a.2 + b.2). Elastic fibers pointed out by red arrows (b.2). Alizarin Red (c.1–c.6) and von Kossa (d.1–d.6, e.1–e.6 and f.1–f.6) stains of cross sections of the pericardium patches P1, P2, P5, P6, P8 and P9 with increasing magnifications: Alizarin Red × 30 (c.1–c.6), von Kossa × 30 (d.1–d.6), von Kossa × 200 (e.1–e.6) and von Kossa × 500 (f.1–f.6).

The principal tissue stains HE and EvG, exemplarily depicted for cross sections of P2 mainly show collagen fibers of the pericardium (Figs. 4a.1–4b.2) traversed by individual elastic fibers (Fig. 4b.2, pointed out by red arrows).

The special stains used to identify mineralization in tissue sections, Alizarin Red and von Kossa, are also depicted in Figure 4. With Alizarin red (Figs. 4c.1–4c.6), the calcified areas appeared stained red, and brown-black at von Kossa staining (Figs. 4d.1–4f.6). Depending on the fluid used, both, calcification onto the materials’ surface and intrinsic calcification within the collagenous matrix occurred in a more or less pronounced manner.

### Fluid-Material-Differentiation-Test

**FIGURE 5:**
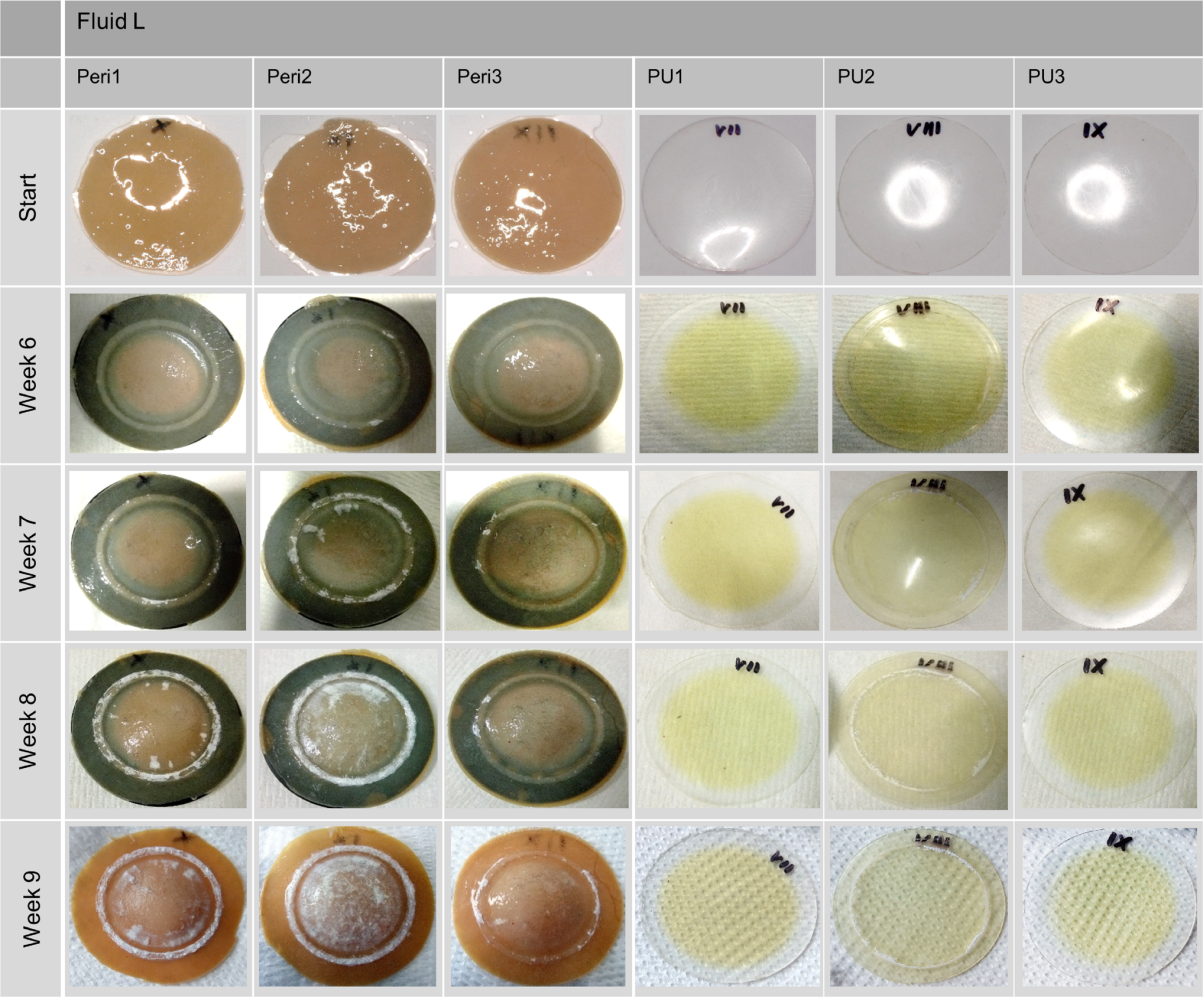
Progression of *in vitro* calcification on pericardium patches (Peri1–Peri3) vs. polyurethane patches (PU1–PU3) conducted in fluid L for 9 weeks (showing week 6–week 9).

The calcification behavior differed distinctly between the two test groups, pericardium and polyurethane test patches. The reference part of fluid L exhibited no precipitation. The pericardium patches showed a recognizable start of calcification at 6 weeks right next to the circular line of fixation. Radial inwards growth of the deposits was observed during the following 3 weeks (Fig. 5). However, two of the polyurethane patches (PU1 + PU3) did not show any calcification over the entire test period. At PU2 a slight fringe of calcification emerged in the space between the fixation rings and the patch even after 4 weeks of testing, which increased during the following 5 weeks (Fig. 5). Of note, here also the fixation rings themselves had strong deposits and the entire compartment showed white precipitate. Even after rinsing the compartment with distilled water after each test week, the snowily precipitate reappeared every week.

#### Structural Analysis of the Deposits

##### XRD

**FIGURE 6:**
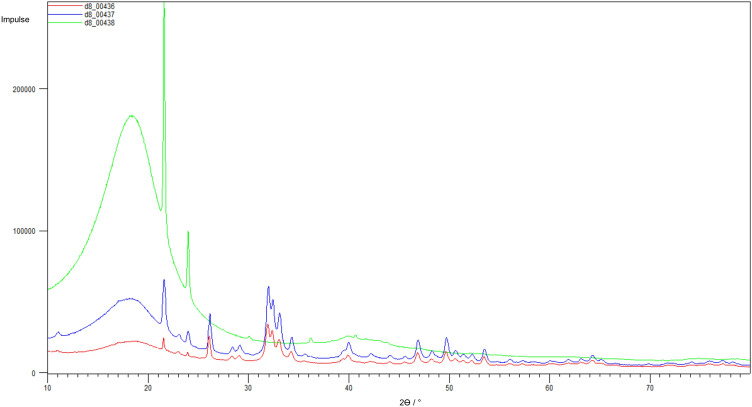
X-ray powder diffractograms of the deposits of the investigated pericardium patches Peri1 (red line) and Peri2 (blue line), the reference spectrum of vaseline (green line).

The structural analysis was conducted from the patches with the most amount of deposits, Peri1 and Peri2. X-ray powder diffractograms of the patch-deposits are depicted in Figure 6. The resulting peak patterns of the deposits of Peri1 and Peri2 were similar (Fig. 6, red line and blue line). Additionally, the reference spectrum of vaseline is shown in Figure 6 (green line) to determine the noise component of the signals of the diffractograms of the patch-deposits. The pericardium patch Peri3 with a little less amount of deposits was provided for the histological examination.

#### Histological Examination

**FIGURE 7:**
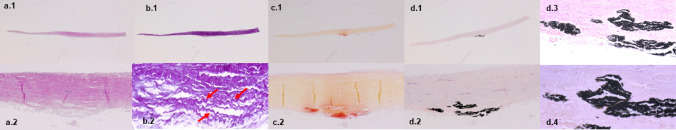
HE (a.1 + a.2), EvG (b.1 + b.2), Alizarin Red (c.1 + c.2) and von Kossa (d.1–d.4) stains of cross sections of the pericardium patch Peri3 with increasing magnifications: HE, EvG, Alizarin red and von Kossa × 30 (a.1, b.1, c.1 and d.1). HE, Alizarin Red and von Kossa × 200 (a.2, c.2 and d.2). EvG × 500 (b.2). Von Kossa × 500 (d.3) and von Kossa × 1000 (d.4).

Cross sections of the calcified area of Peri3 indicated collagen fibers at the principal stains HE and EvG (Figs. 7a.1–7b.2), which were traversed by individual elastic fibers (Fig. 7b.2, pointed out by red arrows). The special stains, especially the von Kossa stain revealed a distinct intrinsic calcification (Figs. 7d.2–7d.4).

## Discussion

To provide a cost- and time-saving method for the assessment of the calcification potential of bioprosthetic heart valves, several attempts towards an accelerated in-vitro model were undertaken. Due to the problem of superficial calcification or spontaneous precipitation, which occurred in the fluids applied,^3^,^10^,^14^ we focused our investigation on the development of a near-physiological non-spontaneously precipitating calcification fluid and the assessment of its calcification behavior in contact with prosthetic materials under dynamic conditions. Calcification behavior includes the initiation, progression, structural appearance and histological localization of the deposits.

In (avital) bioprosthetic valve replacement, which is often preferred to mechanical valve replacement because of the lack of need for lifelong anticoagulation, failure of the valve due to calcification-related stiffening of the leaflets after about 10 to 12 years is a serious problem.^26^ The cause of this phenomenon is attributed to mechanical, chemical and biological processes.^5^ Decades of research in this field has led to various explanations regarding the process and different approaches of valve conditioning. The explanatory approaches and influencing factors are still controversially discussed, e.g. the question whether the initial crystallization process is a purely physical-chemical precipitation of a metastable solution (blood) with possibly subsequent cell and protein involvement, or whether the nucleation itself is already cell-controlled.^5^,^23^,^24^,^27^,^30^ In the healthy organism, a balanced ratio of calcification inducers, (high concentration of mineral ions that lead to metastability of the blood in terms of hydroxyapatite crystallization, additional enzymatic release of inorganic phosphates from organophosphates) and inhibitors (Fetuin-A, pyrophosphate, matrix Gla protein^19^) is assumed, so that ectopic mineralization is prevented.^5^ For the pathological occurrence of calcification, a disturbance of the mechanism of inhibition is assumed, which may be both, systemically caused or induced by potentially calcifying materials. Thus, in bioprosthetic heart valve replacement, it is believed that after glutaraldehyde (GA) fixation of the valve tissue, the dead cells contained therein may act as nucleators of the calcification due to the phosphate groups of their lipid membranes, thus contributing additional inducers into the system.^4^,^5^

### Fluid-Reference-Test of Four Different Calcification Fluids by Dynamic Contact with a Potentially Calcifying Material

As expected from the preliminary fluid study,^13^ the spontaneously precipitating fluids C and E showed a distinct macroscopic calcification of the pericardium patches after 3 to 4 weeks (Fig. 1) whereas the non-spontaneously precipitating fluids revealed no calcification within 4 weeks. Only after raising the calcium concentration in Fluid F, while the ionic product of the fluid was deliberately kept below the solubility product of DCPD^13^(Table 1), calcification of the patches began in the new Fluid L after 6 weeks of the entire test duration. Fluid L itself revealed no spontaneous precipitation. Here, the material influence of the pericardium on the calcification was clearly visible. The fluid alone did not show homogeneous nucleation, but in the presence of the potentially calcifying material heterogeneous nucleation and secondary seed adsorption took place.^3^,^20^,^21^ The calcifying potential of the pericardium may be ascribed to the tissue texture (collagen fibers, elastic fibers, glutaraldehyde treatment, debris of dead cells, roughness). Moreover, the calcification of all calcified patches commenced at the transition from the firmly clamped to the movable area of the patches where the mechanical bending stress applied to the patch material was highest.^25^ Thus an additional material stress is suspected here, which obviously had an influence on the location of calcification initiation. Compared to Fluid L, Fluid G had an even lower calcification potential due to its saturation level (Table 1), which was noticeable in a conspicuous delay in the start and extent of calcification (Fig. 2, P10–P12). Even after 9 weeks of testing only scattered spots of calcification were recognizable, whereby the region of highest mechanical bending stress^25^ was also affected here. In terms of accelerated tests, Fluid L would therefore be preferable, as it fulfills all the necessary conditions (no spontaneous precipitation, almost physiological composition) despite its higher calcification potential. Nevertheless, the use of Fluid G should be further considered in the dynamic testing of bioprosthetic heart valves, as they may have a higher tendency to calcify due to their different load distributions compared to patches. In this case, a fluid with a lower calcification potential could possibly be used to reveal even more detailed differences between different bioprostheses. This will be verified in further tests with bioprosthetic heart valves.

Besides the calcification potential and the macroscopic localization of the occurring calcification, the structural characterization and microscopic or histological localization of the deposits played a decisive role in the assessment of the fluids.

#### Structural Analysis of the Deposits by XRD

**FIGURE 8:**
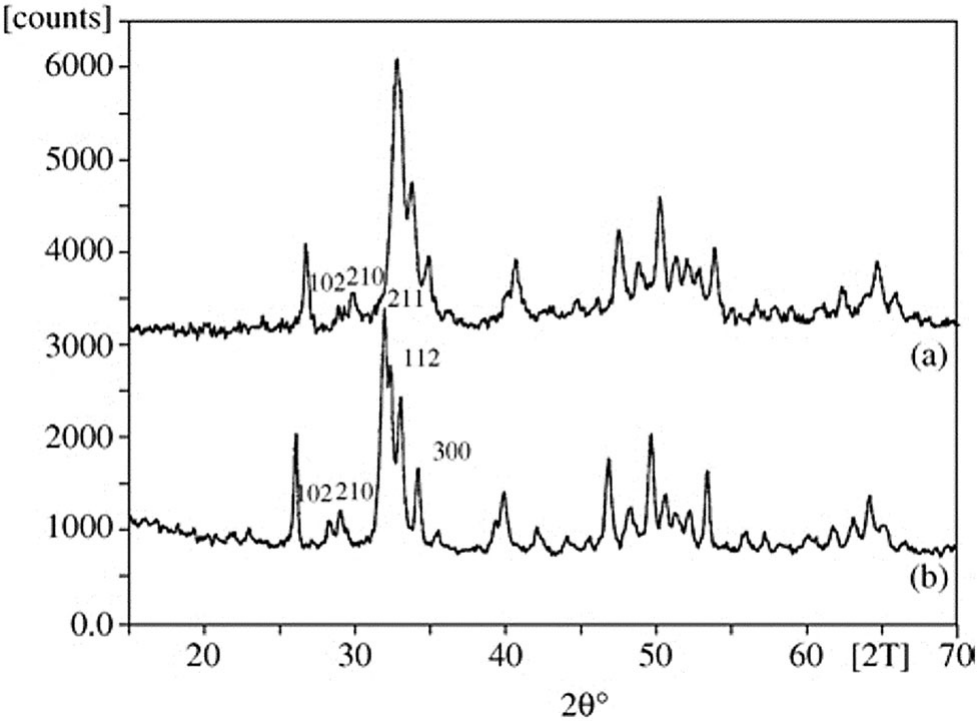
X-ray powder reference diffractograms out of literature^15^ of calcific deposits on human aortic valves (a) and synthetic HAP (b).

According to the comparison with the reference maps for DCPD, OCP and HAP^13^ and the literature diagrams of synthetic apatite and the deposits on a human aortic valve (Fig. 8),^15^ HAP represented the main phase of deposition of all patches (compare Figs. 3a–3e).

Even Fluid C, which in the previous fluid study^13^ produced OCP as the predominant phase (see Fig. 3b), showed HAP as the predominant phase in material contact. Since this fluid is a spontaneously precipitating fluid, it is assumed that in the presence of the pericardium, both homogeneous and heterogeneous nucleation took place, whereby the homogeneously formed nuclei also found attachment sites on the materials’ surface. The fact that HAP was the predominant phase here compared to pure fluid precipitation, could be due to the active involvement of the pericardial collagen textures and residues of charged sidechains in the initiation, orientation and phase transformation of the crystallization process,^17^,^29^ so that HAP formation was favored both thermodynamically and kinetically. According to the literature,^17^ ACP is considered a precursor of collagen mineralization, which can transform into HAP (the thermodynamically most stable phase at pH 7.4 and 37 °C)^8^ both, directly and *via* OCP. The conversion from ACP or OCP to HAP depends on the Ca^2+^-availability and takes place within 24–72 h as stated in literature.^2^,^8^,^28^ In our study, all diffractograms of patch-deposits (Figs. 3b–3e) showed HAP as the predominant phase, suggesting that at the time of sampling the conversion had already taken place, so that the precursor phases could not be identified in the diffractograms. Fluid C itself had the lowest Ca^2+^ concentration of the considered fluids, so that it can be assumed that in the pure fluid study (without heterogeneous catalysis) the conversion of OCP to HAP was delayed or prevented as a function of the lower Ca^2+^-availability, which was exceeded by the material effect in the patch study. Fluid E, which had a distinctly higher Ca^2+^-content, already showed HAP as the predominant phase of the spontaneous precipitates in the fluid study^13^ (Fig. 3c blue line). This certainly supports the theory of Ca^2+^-availability as a controlling factor in OCP/HAP transformation.

#### Histological Examination

All von Kossa stains (Figs. 4d.1–4f.6) of the patch cross sections showed both, calcification on the patch surface and collagen matrix internal intrinsic calcification, which underlines the active participation of the patch material (collagenous fibers) as heterogeneous nucleator.^17^,^29^ The heterogeneous nucleation can take place within the collagen structure as well as on the patch surface. It was remarkable that in the spontaneously precipitating fluids C and E (patches P1, P2 and P5, P6) the calcification along the patch surfaces appeared more pronounced than in the non-spontaneously precipitating fluid L (patches P8 and P9) (see Figs. 4d.1–4e.6). This could probably be ascribed to an additional deposition of the homogeneously nucleated crystals on the patch surfaces, which led to a predominantly superficial calcification.

### Fluid-Material-Differentiation-Test of the Non-spontaneously Precipitating Fluid L with Two Different Materials

As expected for fluid L from the Fluid-Reference-Test, the pericardial patches showed the macroscopic beginning of calcification after week 6 of testing. Comparable to the Fluid-Reference-Test, the calcification of the pericardial patches commenced at the area where the mechanical bending stress due to the clamping was highest^25^ (Fig. 5). The polyurethane patches, in contrast, with the exception of patch PU2 (as described in the results), exhibited no calcification even after 9 weeks of testing. This can be attributed to the fact that polyurethane calcification is a surface phenomenon,^31^,^32^ so that no deposition occurred after testing in the non-spontaneously precipitating fluid. Furthermore, this suggests that the patches had no surface defects, roughness or porosity to the extent necessary to trigger calcification. Even the mechanical stress applied apparently did not cause any surface defects that served as precipitation nuclei. Although patch PU2 was made from the same PU in the same way as the other two patches and was tested under the same conditions, a different picture emerged here. Since the compartment and the fixation rings of patch PU2 revealed a recurrent white deposit infestation, it was assumed that the test compartment or the fixation rings themselves had an unknown contamination that led to the white precipitate, especially since this phenomenon has not been observed in any of our further calcification tests. Therefore, the results of patch PU2 were not considered representative.

Based on the results for the patches PU1 and PU3, it was found that the tested polyurethane in the shape of foils did not represent a heterogeneous calcification nucleator.

#### Structural Analysis of the Deposits by XRD

As the comparison with the deposits of the Fluid-Reference-Test and the reference maps for DCPD, OCP, HAP^13^ as well as the literature diagram (Fig. 8) proved, HAP also formed the main phase of calcifications in the Fluid-Material-Differentiation-Test.

#### Histological Examination

The result of von Kossa staining of the patch cross-section of Peri3 underlines the pronounced occurrence of intrinsic calcification (Figs. 7d.2–7d.4) with heterogeneous nucleation on collagen structures.

### Study Limitations

Since we have deliberately kept the fluid composition simple and have limited it to the essential physiological calcification factors, i.e. we have refrained from adding fluid-side inducers or inhibitors, a possible effect of non-collagenous proteins, as discussed in the literature,^17^,^28^,^29^ could not be investigated. As a further consequence, even systemically acting anti-calcification coatings, as they are partly considered for bioprosthetic heart valves, cannot be sufficiently investigated with the simplified fluid. These would require systemically active fluids based on cell culture medium with the addition of proteins and enzymes, possibly *via* fetal calf serum and addition of suitable cells. The *in vitro* use of these fluids is a challenge, especially because of the need to avoid long-term contamination, and is part of our ongoing studies. For further application in standard *in vitro* tests, the use of such fluids would also represent a cost issue.

Another limitation arose from the fact that we wanted to develop an accelerated test to get results within a reasonable time. The increased mechanical load applied for this purpose led to expansions of the firmly clamped patches, especially the pericardial patches, which are certainly not comparable with deformations under physiological conditions. In order to achieve a more realistic performance under accelerated conditions, further systematic calcification studies with heart valve prostheses that react differently to stress due to their opening and closing behavior need to be conducted. The influence of patch deformation on calcification behavior was not investigated in this study, as this study only focused on evaluating the fluid used, based on its ability to differentiate between different calcification propensities of the tested materials, regardless of what caused these different tendencies.

## Conclusion and Outlook

The fluid reference study allowed us to select the most suitable non-spontaneously precipitating fluid for dynamic *in vitro* calcification tests from four resp. five different fluid compositions. The comparison of the XRD analyses of the *in vitro* calcifications with literature diagrams of synthetic hydroxyapatite and human aortic valve calcifications, revealed HAP as the main phase formed in pericardial contact. The Fluid-Material-Differentiation-Test confirmed the calcification potential of Fluid L and showed its ability to differentiate between materials with different calcification propensities by means of pericardial patches and polyurethane foils. Also in this test, HAP could be confirmed as the main mineral phase of the deposits, as well as the distinct matrix internal intrinsic localization of the deposits, besides a smaller amount near the substrates’ surface.

In further tests, it would be interesting to examine differently pretreated bioprosthetic heart valves in Fluid L instead of patches. It may even be necessary to switch to Fluid G with even weaker calcification potential, since bioprosthetic heart valves may have a higher calcification potential in the dynamic test setting than the patches, due to different load distributions.

A further step in the development of a suitable *in vitro* calcification method will be the adaptation of the fluid to systemic physiological conditions in order to include protein action and possibly cell participation in the calcification model and to create a suitable environment for the assessment of systemically active anti-calcification treatments.

## Abbreviations

ACP: Amorphous calcium phosphate
Ca_T_: Total calcium
DCPD: Dicalciumphosphate-dihydrate
EvG: Elastic Van Gieson stain
HAP: Hydroxyapatite
HE: Hematoxylin and eosin stain
I: Ionic strength
IP: Thermodynamic ionic product
IZKF: Interdisciplinary Center for Clinical Research
K_sp_: Thermodynamic solubility constant
OCP: Octacalcium phosphate
PE: Polyethylene
P_T_: Total phosphate
PU: Polyurethane
PVC: Polyvinyl chloride
*S*_CaP_: Degree of supersaturation
T: Temperature
XRD: X-ray powder diffraction
